# EANM procedural guidelines for radionuclide myocardial perfusion imaging with SPECT and SPECT/CT: 2015 revision

**DOI:** 10.1007/s00259-015-3139-x

**Published:** 2015-08-21

**Authors:** Hein J. Verberne, Wanda Acampa, Constantinos Anagnostopoulos, Jim Ballinger, Frank Bengel, Pieter De Bondt, Ronny R. Buechel, Alberto Cuocolo, Berthe L. F. van Eck-Smit, Albert Flotats, Marcus Hacker, Cecilia Hindorf, Philip A. Kaufmann, Oliver Lindner, Michael Ljungberg, Markus Lonsdale, Alain Manrique, David Minarik, Arthur J. H. A. Scholte, Riemer H. J. A. Slart, Elin Trägårdh, Tim C. de Wit, Birger Hesse

**Affiliations:** 1 Department of Nuclear Medicine, F2-238, Academic Medical Center, University of Amsterdam, Meibergdreef 9, 1105 AZ Amsterdam, The Netherlands; 2 Institute of Biostructures and Bioimaging, National Council of Research, Naples, Italy; 3 Center for Experimental Surgery, Clinical and Translational Research, Biomedical Research Foundation, Academy of Athens, Athens, Greece; 4 Department of Nuclear Medicine, Guy’s Hospital – Guy’s & St Thomas’ Trust Foundation, London, UK; 5 Department of Nuclear Medicine, Hannover Medical School, Hannover, Germany; 6 Department of Nuclear Medicine, OLV Hospital, Aalst, Belgium; 7 Cardiac Imaging, University Hospital Zurich, Zurich, Switzerland; 8 Department of Advanced Biomedical Sciences, University Federico II, Naples, Italy; 9 Nuclear Medicine Department, Hospital de la Santa Creu i Sant Pau, Universitat Autònoma de Barcelona, Barcelona, Spain; 10 Division of Nuclear Medicine, Department of Biomedical Imaging and Image-Guided Therapy, Medical University of Vienna, Vienna, Austria; 11 Department of Radiation Physics, Skåne University Hospital, Lund, Sweden; 12 Heart and Diabetes Center North Rhine-Westphalia, Institute for Radiology, Nuclear Medicine and Molecular Imaging, University Hospital of the Ruhr-University Bochum, Bad Oeynhausen, Germany; 13 Department of Medical Radiation Physics, Lund University, Lund, Sweden; 14 Department of Clinical Physiology and Nuclear Medicine, Bispebjerg Hospital, Copenhagen, Denmark; 15 Department of Nuclear Medicine, Service Commun Investigations chez l’Homme, GIP Cyceron, Caen University Hospital, Caen, France; 16 Radiation Physics, Skåne University Hospital, Malmö, Sweden; 17 Department of Cardiology, Leiden University Medical Center, Leiden, The Netherlands; 18 Department of Nuclear Medicine and Molecular Imaging, University of Groningen, University Medical Center Groningen, Groningen, The Netherlands; 19 Clinical Physiology and Nuclear Medicine, Skåne University Hospital and Lund University, Malmö, Sweden; 20 Department of Clinical Physiology and Nuclear Medicine & PET, Rigshospitalet, University Hospital of Copenhagen, Copenhagen, Denmark

**Keywords:** Guidelines, Myocardial perfusion imaging, Nuclear medicine, Procedures

## Abstract

Since the publication of the European Association of Nuclear Medicine (EANM) procedural guidelines for radionuclide myocardial perfusion imaging (MPI) in 2005, many small and some larger steps of progress have been made, improving MPI procedures. In this paper, the major changes from the updated 2015 procedural guidelines are highlighted, focusing on the important changes related to new instrumentation with improved image information and the possibility to reduce radiation exposure, which is further discussed in relation to the recent developments of new International Commission on Radiological Protection (ICRP) models. Introduction of the selective coronary vasodilator regadenoson and the use of coronary CT-contrast agents for hybrid imaging with SPECT/CT angiography are other important areas for nuclear cardiology that were not included in the previous guidelines. A large number of minor changes have been described in more detail in the fully revised version available at the EANM home page: https://eanm.org/wp-content/uploads/2025/04/2015_myocardial_perfusion.pdf.

## Preamble

The 2015 update of the European procedural guidelines for radionuclide myocardial perfusion imaging (MPI) with single-photon emission computerized tomography (SPECT) was developed on the initiative of the Cardiovascular Committee of the European Association of Nuclear Medicine (EANM) [1]. The guidelines summarize the views of the Cardiovascular Committee of the EANM and reflect recommendations for which the EANM cannot be held responsible. The recommendations should be taken into the context of good practice of nuclear medicine, and they do not substitute for national and international legal or regulatory provisions. The guidelines were brought to the attention of all other EANM Committees and to the National Societies of Nuclear Medicine and have been approved by the EANM.

The present guidelines are based on the guidelines from 2005 [2]. The 2015 update includes all aspects of SPECT imaging from gating to hybrid imaging, but does not include myocardial perfusion evaluated by positron emission tomography (PET), which will be updated in a separate guideline. The 2015 guideline also updates relevant sections of the 2008 guidelines on radionuclide imaging of cardiac function [3] and the 2011 joint position statement on hybrid cardiac imaging [4].

The 2015 EANM guidelines on myocardial perfusion imaging (MPI) include the following sections:

1. Patient information and preparation
2. Radiopharmaceuticals and CT contrast agents
3. Injected activities, dosimetry, and radiation exposure
4. Stress tests
5. Instrumentation
6. Imaging protocols
7. Image acquisition
8. Quality control of instrumentation and image acquisition
9. Reconstruction methods
10. Attenuation and scatter correction
11. Data analysis of regional perfusion imaging
12. Data analysis of left ventricular function
13. Data analysis of hybrid imaging
14. Reports and image display.

All sections have been updated and a lot of details were revisited. The present paper highlights the major changes presented in the updated 2015 guidelines and focuses on the selective coronary vasodilator regadenoson, recent developments on calculated radiation exposure [i.e., new International Commission on Radiological Protection (ICRP) models, dedicated cardiac SPECT cameras and hybrid systems], recent developments in instrumentation (i.e., dedicated cardiac SPECT and cardiac hybrid imaging systems) and coronary CT-contrast agents. All 14 sections are described in more detail in the fully revised version available at the EANM home page [1].

The EANM guidelines are intended to present information specifically adapted to European practice, based on evidence from original scientific studies or on previously published guidelines [i.e., for the 2015 MPI guidelines as well as for the extract presented below: on national or European guidelines for MPI, on the European Society of Cardiology (ESC), and on the American College of Cardiology (ACC) / American Heart Association (AHA) / American Society of Nuclear Cardiology (ASNC) guidelines]. In case of a lack of published evidence, opinions are based on expert consensus and are indicated as such. Where more than one solution seems to be practised, and none has been shown to be superior to the others, the committee has tried to specifically express this state of knowledge.

## The selective coronary vasodilator regadenoson

### Mechanism of action

Vasodilators induce myocardial hyperaemia mediated by adenosine receptors independent of myocardial oxygen demand. Only A_2A_ receptors induce coronary vasodilation, and hereby a fourfold to fivefold increase in myocardial blood flow in healthy coronary vessels. Besides, the A_2A_ receptor adenosine also stimulates A_1_, A_2B_, and A_3_ adenosine receptors, which provoke the adverse effects [5]. Regadenoson is a selective stimulator of the A_2A_ receptor with minimal or no stimulation of the other adenosine receptor subtypes [5]. Dipyridamole increases the tissue levels of adenosine by preventing the intracellular reuptake and deamination of adenosine [5]. Vasodilatation results in a modest increase in heart rate and most often a modest decrease in both systolic and diastolic blood pressures.

For regadenoson, as with the other vasodilators, caffeine-containing beverages (coffee, tea, cola, etc.), foods (chocolate, etc.), some medicaments (e.g., pain relievers, stimulants and weight control drugs) and methylxanthine-containing medications that antagonise vasodilator action must be discontinued at least 12 h before vasodilator stress and at least five half-lives before for long-acting methylxanthines. Dipyridamole or medications containing dipyridamole should be interrupted for at least 24 h. Pentoxifylline and clopidogrel need not be discontinued. Ticagrelor, a direct-acting P2Y12-adenosine diphosphate receptor blocker, has been shown to significantly raise adenosine plasma levels so that more frequent and more severe adverse effects in adenosine and dipyridamole stress testing are likely [6, 7]. Interactions with regadenoson have not yet been studied. So far there are still no recommendations for dose modification of vasodilators in stress testing for patients receiving ticagrelor.

### Indications

**Fig. 1:**
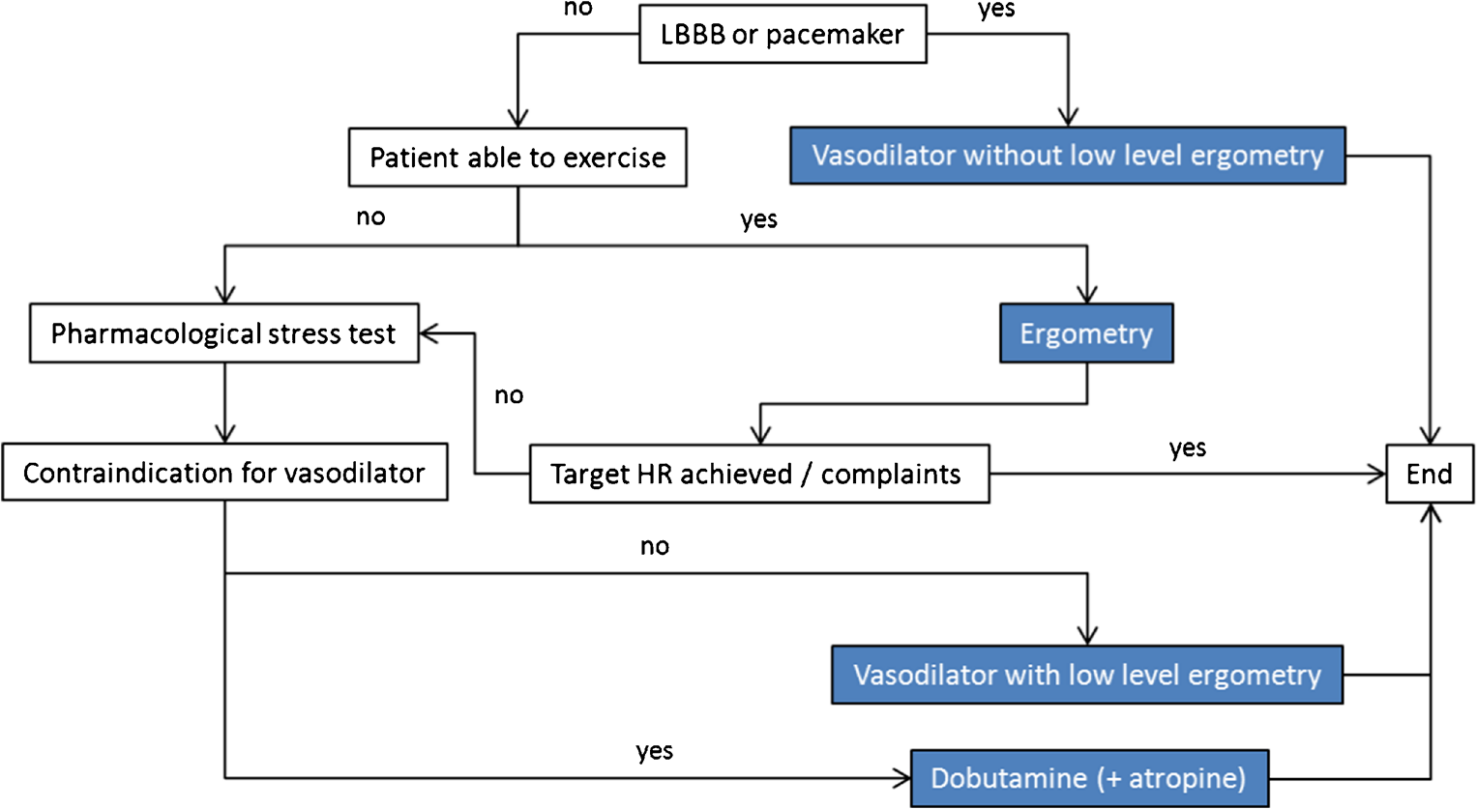
Selection of stress test modality. Except for patients with left bundle branch block (LBBB) or ventricular paced rhythm, consider combining pharmacological vasodilatory stress with low-level exercise according to the ability of the patient to exercise. In case of pharmacological stress with dobutamine but without adequate heart rate response, consider to add atropine

The indications are the same as for exercise MPI, but refer to patients who are not able to or who are expected to be unable to achieve ≥ 85 % of maximal age-predicted heart rate during exercise. Vasodilators (without exercise) should be preferred to exercise in cases of left bundle branch block or ventricular paced rhythms (Fig. 1). Considering diagnostic performance of MPI, there is no significant difference among the stress agents and modalities [8–10].

### Combination with low-level exercise

Low-level exercise can be performed routinely in conjunction with vasodilator tests. Low-level exercise significantly reduces vasodilator-induced side effects (flushing, dizziness, nausea, headache, hypotension) and improves image quality due to lower bowel activity and higher target-background ratio. Accordingly, if possible, low-level exercise is recommended in combination with vasodilator stress testing [11, 12].

### Regadenoson dose

**Fig. 2:**
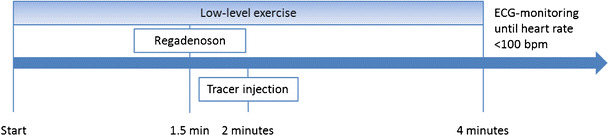
Timeline of regadenoson plus low-level exercise testing. *BPM* beats per minute

Regadenoson is given as a slow bolus over 10 s followed by a 10 s flush of 5–10 ml NaCl 0.9 %, and 10–20 s later the radiopharmaceutical is injected (Fig. 2). Regadenoson is administered independent of patient weight in a dose of 0.4 mg in 5 ml.

### Regadenoson safety profile

Compared to adenosine, regadenoson is associated with a lower incidence of chest pain, flushing, and throat, neck, or jaw pain, a higher incidence of headache and gastrointestinal discomfort and a lower combined symptom score in nearly all subgroups [13]. The safety of regadenoson was also studied in two randomized, double-blind, placebo-controlled crossover trials in patients with mild to moderate asthma and moderate to severe chronic obstructive pulmonary disease (COPD), respectively. Although dyspnoea was reported in the regadenoson groups compared to none in the placebo groups, there was no relation with a decline in forced expiratory volume in 1 s (FEV1) [14, 15]. Thus, regadenoson can also be used in patients with mild asthma and COPD. In summary, despite their selectivity to the A_2A_ receptor, side effects related to activation of the other adenosine receptors continue to occur, albeit at a lower frequency and with less severity and duration compared to the selective adenosine agonists.

A less frequent but important side effect of regadenoson is a low but significant increase in incidence of seizures. The exact incidence is unknown and the pathophysiological mechanism behind this increased incidence is not yet clear. Regadenoson may lower seizure threshold, and aminophylline should not be used in cases of seizures associated with regadenoson. These seizures may be of new onset, or may be recurrences. In addition, some seizures are prolonged and may require urgent anticonvulsive management. It is recommended that during the initial triaging of patients, they should be asked about a history of seizures [16–18].

According to the available limited data, the absolute risk of a transient ischaemic attack (TIA) and cerebrovascular accident (CVA) associated with regadenoson administration appears to be small and may not be different from other stress agents [19]. However, based on recent advice from the European Medicines Agency, a Direct Healthcare Professional Communication updated the regadenoson product information [20]:

- As aminophylline increases the risk of prolongation of a regadenoson-induced seizure, it should therefore not be administered solely for the purpose of terminating a seizure induced by regadenoson
- Delay regadenoson administration in patients with elevated blood pressure until the latter is well controlled
- There is a rare but undesirable risk of a TIA and CVA associated with regadenoson administration.

## Radiation exposure: new ICRP models

The ICRP has adopted new phantoms for absorbed dose calculations, new weighting factors, and new bio-kinetic models [21–23]. Using these new models, the effective doses per administered unit of activity to adults are [24]:

- ^99m^Tc-tetrofosmin (stress and rest): 0.0058 and 0.0063 mSv/MBq, respectively
- ^99m^Tc-sestamibi (stress and rest): 0.0066 and 0.0070 mSv/MBq, respectively
- ^201^Tl-chloride (redistribution): 0.102 mSv/MBq.

These values are approximately 20 % lower than the values calculated using the previous methods.

### Activity amounts to inject

Camera systems based on new technology (e.g., cadmium-zinc-telluride (CZT)-cameras) have improved count sensitivity. This improved sensitivity can be used to either reduce the amount of activity accordingly, or decrease image acquisition duration [25–29]. The activities to be administered for paediatric patients should be modified according to the recommendations of the EANM [30].

### Hybrid systems

If the MPI is combined with CT for attenuation, calcium scoring (CACS) or coronary CT angiography (CCTA), an additional dose is given to the patient. For an attenuation correction CT, an additional 0.5–1.0 mSv is given. Absorbed doses from CACS and CCTA depend on the system and imaging protocol used, and for CACS can be estimated to be < 1 mSv. The absorbed dose for CCTA can be estimated to be between 2–5 mSv using commonly available single-source 64-slice CT scanners with a prospectively ECG-triggered, step-and-shoot acquisition protocol [31, 32]. The latest generation dual-source or 256-slice and 320-slice single source CT scanners enable even absorbed doses < 1 mSv [33, 34].

## Instrumentation: dedicated cardiac systems

Since the publication of the first version of this guideline, some significant advances in technology for cardiac imaging have been made [35–37]. In new-generation, dedicated cardiac, ultrafast-acquisition scanners, multiple detectors surrounding the patient simultaneously image the heart. Variations in these newly designed, dedicated cardiac scanners comprise the number and type of scanning or stationary detectors and the use of NaI, CsI, or CZT solid-state detectors. They all have in common the potential for a fivefold to tenfold increase in count sensitivity at no loss of resolution, resulting in the potential for acquiring a scan in 2 min or less if the patient is injected with a standard activity. Some of this gain in sensitivity can be traded for a linear reduction in the injected activity to reduce the patients’ exposure to radiation. With an ultrafast camera with a tenfold increase in sensitivity using conventional radiopharmaceutical activity, the dose could be reduced by half and a fivefold increase in sensitivity would still be maintained [36].

Dedicated systems have emerged based on traditional scintillation detector technology, equipped with smaller crystals and thus having more compact design, as well as truly innovative cameras with a completely novel detector technology. Pixelated, solid-state detectors made of CdZnTe (an alloy of cadmium telluride and zinc telluride, CZT) offer better energy resolution and a virtually linear count rate response. As CZT replaces both the scintillation crystal and the attached photomultiplier tubes, the detectors are very compact. This has thus far been exploited in two different, commercially available cameras. The D-SPECT® (Spectrum Dynamics) utilises nine small rectangular detectors placed along a 90° arc. Each detector rotates around its own axis. All detectors together register photons from an area comparable to a traditional 180° acquisition. The other CZT system (the Discovery NM530c by GE Healthcare) uses a stationary, multiple-pinhole design with 19 holes, each with its own CZT detector. The collimators are arranged such that the area of the chest including the heart can be imaged. Clinical evaluations of both systems have demonstrated performance similar to that of traditional systems, but with shorter imaging times or lower administered activities [36]. In a recent study, the D-SPECT® system was compared to a conventional SPECT camera, showing improved image quality, comparable incidence of extracardiac activity, and achieving a reduction in absorbed dose to 1 mSv for a single injection [38].

Another variation from traditional design is the upright patient position offered in a number of dedicated systems. While more comfortable for the patient, the effect of posture on organ position must be considered when interpreting the images [39, 40]. Some cameras systems (e.g., Digirad Cardius) go one step further and employ a rotating patient chair instead of a rotating gantry. Additionally, CsI(Tl) detectors allow for an optional X-ray-based attenuation correction method. Another variation of the upright design (the CardiArc system) is based on three stationary arc-shaped crystals combined with a moving aperture, providing a similar function as a collimator. A problem with many different, dedicated cardiac systems is the scarcity of publications about their clinical validity [36].

An upgraded technology (IQ-SPECT, Siemens Healthcare) uses a conventional multi-purpose SPECT system with a radially oriented, symmetric, cardio-focal collimator that is characterised by a radially increasing focal spot distance. The proprietary reconstruction algorithm matches the collimators’ spatially varying sensitivity profile. Reductions in acquisition time with no loss in image quality are claimed, but the clinical evidence is limited.

## Instrumentation: SPECT/CT hybrid systems

The SPECT detectors in most SPECT/CT systems do not differ in any significant way from those of stand-alone SPECT systems. The SPECT sub-systems are typically large field of view (FOV), variable-angle, dual detector system. For hybrid imaging systems, the CT configuration can be a low-resolution CT (non-diagnostic CT) or a multi-detector-row CT with slices ranging from 2 up to 64. Any of these systems can be used for attenuation correction of MPI. For CACS, at least 4-slice CT is required, but ≥ 6-slice recommended. For CCTA, at least a 16-slice scanner is required, but a ≥ 64-slice multidetector-row CT is recommended, with an imaging capability for slice width of 0.4–0.6 mm and temporal resolution of 500 ms or less; ≤ 350 ms is preferred [41, 42].

In general, it has to be considered that CT imaging is much faster than SPECT, where the heart position is averaged over the complete acquisition time (5–20 min), so that mis-registration artefacts can occur. As a consequence, free-breathing and end-expiratory breath-hold protocols during CT scanning are preferred over inspiration breath-hold protocols when the CT scan is performed for attenuation correction only [43]. General guidelines for CT-based transmission imaging for SPECT are listed in Table 1 [44].

**Table 1:** General guidelines for CT-based transmission imaging for SPECT [44]

CT parameter	General principle	Effect on patient absorbed dose
Slice collimation	Collimation should approximate slice thickness of SPECT (e.g., 4–5 mm)	Thinner collimation often less dose efficient
Gantry rotation speed	Slower rotation helps blurring cardiac motion (e.g., 1 s/revolution or slower)	Increased radiation with slower gantry rotation
Table feed per gantry rotation (pitch)	Pitch should be relatively high (e.g., 1:1)	Inversely related to pitch
ECG gating	ECG gating is not recommended	Decreased without ECG gating
Tube potential	80–140 kVp is used, depending on manufacturer specification	Increases with higher kVp
Tube current	Because scan is acquired only for attenuation correction, low tube current is preferred (10–20 mA)	Increases with higher mA
Breathing instructions	End-expiration breath-hold or shallow free breathing is preferred	No effect
Reconstructed slice thickness	Thickness should approximate slice thickness of SPECT (e.g., 4–7 mm)	No effect

For attenuation correction of perfusion SPECT studies, separate CT scans should be performed for the rest and stress MPI studies, even if recent studies demonstrate that the CT transmission scans are interchangeable in specific clinical settings [45]. Other recent studies have demonstrated that the CT scan can be used to approximate the extent of coronary calcification [46]. This approach, however, is less accurate as compared to dedicated calcium scans due to missing correction of the coronary motion and a much lower photon density. Otherwise, the coronary artery calcium score CT scan can be used for attenuation correction, but with the limitation that this scan may not register adequately with SPECT due to different acquisition time points [47, 48]. These are topics of ongoing research and software developments.

For detailed acquisition protocols of the coronary artery calcium scan and CCTA we refer to the joint position statement by the EANM, the ESCR and the European Council of Nuclear Cardiology [4].

## Coronary CT contrast agents

The use of contrast media for cardiac imaging is increasing as hybrid cardiac SPECT/CT and PET/CT, as well as coronary CT angiography and cardiac MRI, become more widely used [4].

For coronary CT angiography (CCTA), an intravenously injected contrast agent is needed. In general, these contrast agents are iodine-based and due to the relatively high attenuation coefficient of iodine, therefore result in high contrast between organs with and those without contrast. Coronary CT in combination with ECG-gating and a contrast agent permits visualization of the coronary artery lumen and detection of coronary artery stenoses [49, 50]. At the moment, there are four classes of contrast media available for clinical use: high osmolar ionic monomers, low osmolar non-ionic monomers, low osmolar ionic dimers and iso-osmolar non-ionic dimers. The contrast media are provided at various iodine concentrations and have different biochemical properties (viscosity, osmolarity, hydrophilic behaviour, ionic content and pH).

In general, the injection protocol should follow vendor recommendations dependent on the specific contrast agent and CT used. For coronary imaging in general, a contrast agent with a high concentration of iodine is used (300–400 mg/ml) to ensure adequate opacification of the small coronary arteries [51]. In total, approximately 60–90 ml of contrast agent is injected at an injection speed of 4–6 ml/s. Often the bolus is split between a first bolus of pure contrast and then a bolus with a mix of contrast and saline, to reduce streak artefacts from contrast enhancement of the vena cava and right side of the heart. Otherwise, the contrast bolus is followed by a saline flush of 40–70 ml. With the newest generation of CT scanners, a smaller contrast bolus with lower iodine concentration is likely sufficient to obtain good contrast enhancement of the coronary arteries [52].

Two contrast timing techniques are available to start the CCTA acquisition, based on the arrival of contrast in the aorta: the bolus tracking and the bolus timing technique. Bolus tracking involves a series of axial low-dose images at 2 s intervals to track the arrival of the bolus of contrast material in the aorta. The CCTA is initiated when the contrast enhancement of the aorta reaches a certain predefined level, e.g., 100 Hounsfield units (HU). The bolus timing technique involves an extra low-dose scan acquisition of a single slice prior to the CCTA acquisition. Here, a small contrast bolus and saline flush are injected to determine the contrast arrival interval. The time between the start of the contrast injection and the arrival of contrast bolus in the aorta is used as the scan delay for the actual CCTA [4, 49].

### Contraindications

Contraindications for iodine contrast can be divided into absolute and relative contraindications [53, 54]. Absolute contraindications include myasthenia gravis, mastocytosis, and post-thyroid carcinoma when follow-up with ^131^I imaging or ^131^I therapy is planned within 6 months of the CCTA. Relative contraindications are known contrast allergy, planned thyroid scan, and multiple myeloma.

The most frequent problem is related to renal failure: The clinical benefit of using estimated glomerular filtration rate (eGFR) or calculated creatinine clearance in assessing pre-procedural, contrast induced nephrotoxicity (CIN) risk in patients with stable renal function is uncertain, because much of the knowledge comes from studies that used only serum creatinine measurements. The threshold values at which different clinical actions should be taken (e.g., active IV hydration, avoidance of contrast medium administration) are neither proven nor generally agreed upon for either serum creatinine measurement or calculated creatinine clearance. In addition, the accuracy of these formulae has only been validated in the patient population for whom they were developed. The following is a suggested list of risk factors that may warrant pre-administration serum creatinine screening in patients who are scheduled to receive intravascular iodinated contrast medium. This list should not be considered definitive [55]:

- Age > 60
- History of renal disease, including dialysis, kidney transplant, single kidney, renal cancer, renal surgery
- History of hypertension requiring medical therapy
- Diabetes.

Metformin does not confer an increased risk of CIN. However, metformin can very rarely lead to lactic acidosis in patients with renal failure. In case of reduced renal function (i.e., estimated glomerular filtration rate < 45 ml/min/1.73 m^2^, or < 60 ml/min/1.73 m^2^ in the presence of diabetes or ≥ 2 risk factors for contrast nephropathy), alternative imaging strategies should be considered. However, when no alternatives are available, adequate hydration is the major preventive action against CIN.

If intravenous contrast agent is going to be administered, metformin should be discontinued at the time of the procedure and withheld for 48 h after the procedure. If the risk of nephrotoxicity is high, metformin can be reinstituted only after renal function has been re-evaluated and found to be normal. If the risk of nephrotoxicity is low, metformin can be re-instituted without the need for renal function assessment. An alternative glucose-controlling drug should be considered during this time [56].

### Adverse events

Strategies to reduce the risk in non-acute, contrast medium induced, adverse reactions include the prophylactic use of oral anti-histamines and corticosteroid tablets. A classification system, stratifying adverse events due to iodinated contrast media by severity and type, is presented below [55, 57, 58]:

- *Minor* Signs and symptoms are self-limited without evidence of progression. Mild reactions include: pruritus, nausea and mild vomiting, diaphoresis
- *Moderate:* Signs and symptoms are more pronounced and commonly require medical management. Some of these reactions have the potential to become severe if not treated. Moderate reactions include: facial oedema, faintness, severe vomiting, urticaria, bronchospasm
- *Severe:* Signs and symptoms are often life-threatening and can result in permanent morbidity or death if not managed appropriately. Severe reactions include: laryngeal oedema, pulmonary oedema, respiratory arrest, hypotensive shock, convulsions, cardiac arrest
- *Delayed:* Skin rash, thyrotoxicosis, kidney dysfunction.

The frequency of allergic-like and physiologic adverse events related to the intravascular administration of iodinated contrast media is low and has decreased considerably with changes in usage from ionic, high-osmolality contrast agents (HOCA) to non-ionic, low-osmolality contrast agents (LOCA). Historically, acute adverse events occurred in 5–15 % of all patients who received HOCA. Many patients receiving intravascular HOCA experienced physiologic disturbances (e.g., generalized warmth, nausea, or emesis), and this was often documented as a contrast reaction. HOCA are now rarely or never used for intravascular purposes, because of their greater adverse event profile compared to LOCA. The reported overall acute adverse reaction rate (allergic-like + physiologic) for non-ionic LOCA (i.e., iohexol, iopromide, or iodixanol) ranges from 0.2 %, 0.6 % to 0.7 % [59–61]. Serious acute reactions to iv. LOCA are rare, with a historical rate of approximately four in 10,000 (0.04 %) [62].

## Reconstruction methods

Myocardial perfusion images are the result of a complex reconstruction process. Although sophisticated reconstruction methods are available, including correction for motion, attenuation and scatter correction, these software tools cannot produce “miracles”. It is therefore important to achieve optimal quality of the raw data by selecting the proper matrix size, angular sampling, zoom factor, patient-to-camera distance, energy window settings, and assuring that the camera is properly tuned and maintained through regular quality control procedures. In addition, the acquired projection data should be checked for motion and the presence of high extra-cardiac uptake. This should be done before the patient leaves the department and before reconstruction is commenced.

Camera vendors and various third party companies provide reconstruction software that implement iterative reconstruction based on Ordered Subset Expectation Maximisation or maximum likelihood expectation maximization (OS-EM/ML-EM). The advantage of these algorithms over traditional filtered-back projection is that information about the camera, patient and radiopharmaceutical can be exploited to reconstruct better images. CT images can be incorporated for estimation of attenuation and scatter; the collimator-detector-response can be modelled and used for resolution recovery; noise can be compensated by modelling the underlying characteristics of the decay process.

These reconstruction methods can achieve enhanced image quality that may be traded against shorter acquisition times or reduced administered activity. Fundamental to all these algorithms is the correct choice of user-selectable parameters (typically number of iterations and subsets, regularisation, and filter parameters). Inadequate parameters most likely lead to insufficient image quality and artefacts. As implementations vary considerably across vendors, it is not possible to transfer settings between camera systems without prior validation.

Currently, all major vendors offer the possibility to include resolution recovery (also called count recovery) in the OS-EM/ML-EM algorithm. The increased reconstructed resolution and lower noise allow for slightly lower count statistics (hence lower injected activity) or shorter scan times [35]. However, such techniques require careful testing against phantom studies performed with validated hardware and software.

## Data analysis of hybrid imaging

**Fig. 3:**
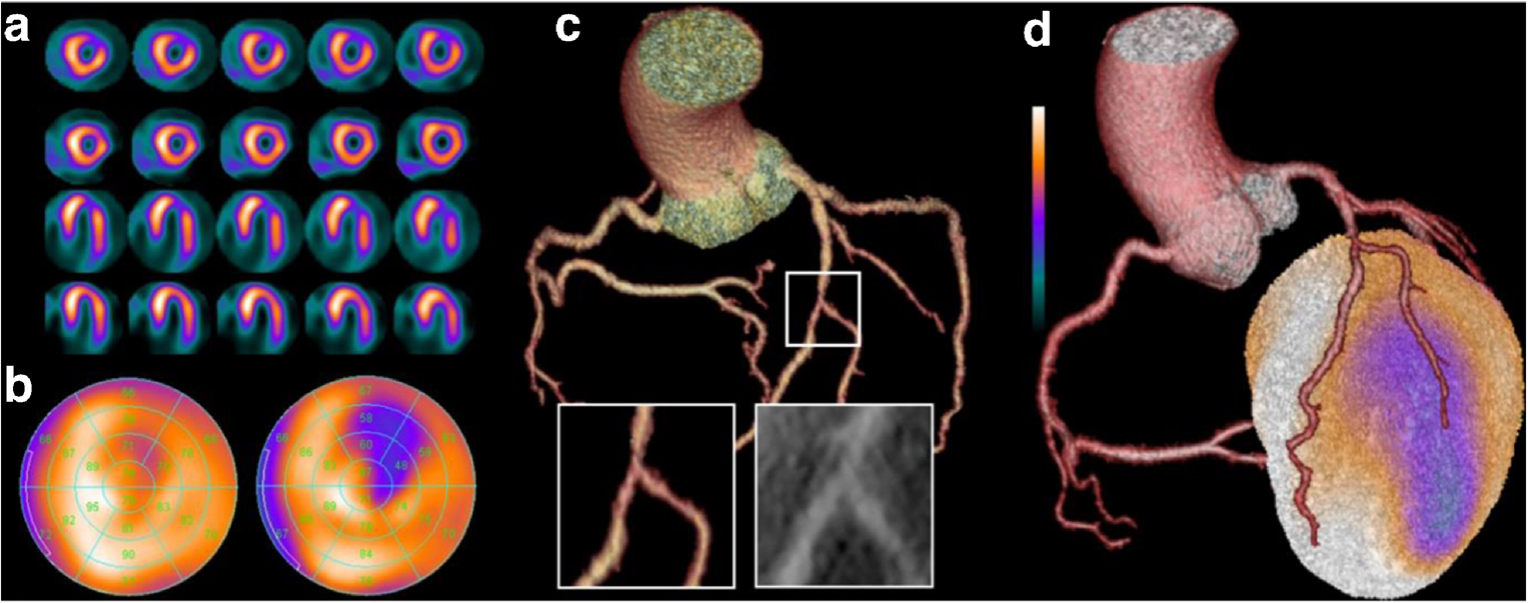
Coronary artery territories in a 17-segment model Myocardial perfusion SPECT, coronary computed tomography angiography (CCTA), and fused hybrid SPECT/CCTA of a 43-year-old male patient with presenting symptoms of typical angina. Myocardial perfusion SPECT documents a reversible perfusion defect in short axis and horizontal long axis slices (**a**) at rest (*bottom rows*) and stress (*top rows*). The corresponding polar plots (**b**) at rest (*left plot*) and stress (right plot) clearly depict the extent of the ischaemic area in the anterolateral wall. CCTA (**c**) shows an intermediate stenosis (i.e., 50–70 % luminal narrowing) due to non-calcified plaques in the middle/distal left anterior descending artery at the level of the second diagonal branch bifurcation. Fused hybrid SPECT/CCTA (**d**) reveals that the anterolateral ischaemia corresponds with the vascular territory of the second diagonal branch, while the stenosis in the left anterior descending artery (LAD) does not cause any ischaemia

Hybrid imaging is defined as the combination and fusion of two data sets by which both modalities significantly contribute to image information [63]. Typically, hybrid imaging is synergistic, i.e. more powerful than the sum of its parts, as it provides information beyond that achievable with either data set alone, leading to improved sensitivity and specificity (Fig. 3).

The term hybrid imaging is not valid for the combination of nuclear cardiac imaging with X-ray-based AC where the (low-dose) CT images do not provide independent information, but only contributes to image quality improvement of the other modality (SPECT) [4].

### Hardware and software requirements

Dedicated workstations capable of two-dimensional and three-dimensional displays of CCTA and SPECT data are a basic requirement for hybrid data analysis. Aside from projection of standard views of SPECT data, the combination of hardware and software has to offer capabilities for volume rendering of stress and rest SPECT data sets (uncorrected and attenuation corrected), common reconstruction and reformatting techniques for CCTA data [including transaxial stacks, multiplanar (curved) reformations, maximum intensity projections and volume-rendering] [64], and automatic or manual image co-registration and fusion. If non-contrast enhanced CT data is acquired for AC of SPECT, the software should offer computation of coronary artery calcium scores.

### Integration of myocardial perfusion SPECT with CCTA

The incremental value of hybrid cardiac imaging arises from spatial co-localization of a myocardial perfusion defect with a subtending coronary artery. Traditionally, this process is performed by mental integration of a standard myocardial segmentation model that allocates each segment to one of the three main coronary arteries [65].

Notably, however, there is a substantial inter-individual anatomical variability of coronary arteries. Therefore, the so-called standard distribution of myocardial perfusion territories does not correspond with the patients’ individual anatomy in more than half of the patients [66]. Most frequently, the left circumflex artery segments are erroneously assigned to the right coronary artery territory, and standard left anterior descending artery segments are erroneously assigned to the left circumflex territory [67]. True hybrid cardiac imaging and data analysis using volume rendered, co-registered, and fused SPECT and CCTA data sets should be preferred over sole side-by-side analysis because of accurate segmental assignment to coronary artery territories and documented incremental value [68–70].

Fused images of myocardial perfusion SPECT and CCTA can be obtained using data sets acquired on a hybrid scanner (SPECT/CT) or via co-registration of images obtained from separate stand-alone scanners. Software-based automated registration is accurate and fast [70]. However, automated co-registration of data from cardiac imaging is more challenging than that from other body areas. Errors may arise from cardiac motion, breathing motion, and cardiac contraction [71]. Additionally, different anatomic features are depicted by the two modalities rendering automatic object recognition and image registration difficult, particularly if large perfusion defects are present in SPECT images. Accurate registration, however, is of utmost importance to accurately allocate subtending coronary arteries to areas with radiotracer uptake. Therefore, review of the registration is mandatory and manual correction must be performed if needed.

### Data analysis

The combined diagnostic information from myocardial perfusion SPECT imaging and CCTA is complementary: the negative predictive value of CCTA to exclude CAD is close to perfect (Fig. 3) [49, 72, 73]. However, specificity and positive predictive value of CCTA for identification of haemodynamically significant luminal narrowing have been documented to be less robust. CCTA is associated with a general overestimation of the severity of coronary artery stenosis and difficulties to differentiate between slight differences in stenosis severity. SPECT offers complementary diagnostic information as to the haemodynamic significance of coronary artery lesions detected by CCTA. Also, uninterpretable segments on CCTA due to strong calcifications or artefacts can be well studied with SPECT. By contrast, CCTA adds to the diagnostic value of SPECT through documentation of multivessel disease (with possible balanced ischaemia not detectable by semi-quantitative SPECT analysis) or diagnosis of subclinical atherosclerosis.

Interpretation of CCTA studies is beyond the scope of this document and is covered in detail in the respective guidelines of the Society of Cardiovascular Computed Tomography [64]. In brief, CCTA should be first reviewed in transaxial images, complemented by multiplanar reformations to better visualize suspected lesions. Diagnostic conclusions are not based on three-dimensional, volume-rendered CCTA representations.

By contrast, in hybrid imaging, a panoramic three-dimensional view is offered by integrating volume-rendered CT data with the perfusion information from SPECT. This comprehensive information improves both the identification of the culprit vessel and the diagnostic confidence for categorizing intermediate lesions and equivocal perfusion defects, thus optimizing management decisions.

Incidental cardiac and extracardiac CT findings are not uncommon [74]. Although most such findings are negligible, some may occasionally be of clinical relevance. Thus, it is recommended that images are additionally screened by a physician fully trained in CT readings, including non-contrast enhanced CT scans for attenuation correction and scouts.

On a practical level, this concept requires careful coordination of the readout between nuclear physicians and radiologists, and the final discussion with the cardiologist. The benefit of such an interdisciplinary approach is that those fully trained in the specific modalities would interpret the images jointly, thus providing a high-quality result covering all aspects of hybrid SPECT/CCTA image acquisition.

The integration of coronary anatomy by CCTA and (quantitative) documentation of the ischaemic burden through SPECT allows effective identification of: 1) patients with CAD benefitting from optimal medical therapy versus those who should undergo coronary revascularization, 2) the culprit stenosis in patients with multiple coronary artery lesions, thereby guiding clinicians on the appropriate method of revascularization, 3) patients with subclinical coronary atherosclerosis where more aggressive prevention may be indicated, and 4) patients with normal coronary arteries who can safely be deferred from any further cardiac testing.

In summary, the complimentary anatomic and functional information provided by hybrid SPECT/CCTA imaging has been demonstrated to confer added diagnostic value in CAD detection [67, 69, 70, 75, 76] and to effectively stratify risk and predict outcomes [77, 78], guide patient management [79], and to contribute to optimal downstream resource utilization [80].

## References

[1] Verberne HJ, Acampa W, Anagnostopoulos C, Ballinger J, Bengel F, De Bondt P, et al. 2015 updated EANM procedural guidelines for radionuclide myocardial perfusion imaging with SPECT and SPECT/CT. https://eanm.org/wp-content/uploads/2025/04/2015_myocardial_perfusion.pdf. 2015. Accessed 7 July 2015. 10.1007/s00259-015-3139-x PMC4589547 26290421

[2] Hesse B, Tagil K, Cuocolo A, Anagnostopoulos C, Bardies M, Bax J, et al. EANM/ESC procedural guidelines for myocardial perfusion imaging in nuclear cardiology. Eur J Nucl Med Mol Imaging. 2005;32(7):855–97. 15909197 10.1007/s00259-005-1779-y

[3] Hesse B, Lindhardt TB, Acampa W, Anagnostopoulos C, Ballinger J, Bax JJ, et al. EANM/ESC guidelines for radionuclide imaging of cardiac function. Eur J Nucl Med Mol Imaging. 2008;35(4):851–85. 18224320 10.1007/s00259-007-0694-9

[4] Flotats A, Knuuti J, Gutberlet M, Marcassa C, Bengel FM, Kaufmann PA, et al. Hybrid cardiac imaging: SPECT/CT and PET/CT. A joint position statement by the European Association of Nuclear Medicine (EANM), the European Society of Cardiac Radiology (ESCR) and the European Council of Nuclear Cardiology (ECNC). Eur J Nucl Med Mol Imaging. 2011;38(1):201–12. 20717824 10.1007/s00259-010-1586-y

[5] Zoghbi GJ, Iskandrian AE. Selective adenosine agonists and myocardial perfusion imaging. J Nucl Cardiol. 2012;19(1):126–41. 22130964 10.1007/s12350-011-9474-9

[6] Bonello L, Laine M, Kipson N, Mancini J, Helal O, Fromonot J, et al. Ticagrelor increases adenosine plasma concentration in patients with an acute coronary syndrome. J Am Coll Cardiol. 2014;63(9):872–7. 24291273 10.1016/j.jacc.2013.09.067

[7] Voci P, Pizzuto F. Coronary flow reserve with a turbo: a warning for the use of adenosine as a provocative test in patients receiving ticagrelor? J Am Coll Cardiol. 2014;63(9):878–9. 24291275 10.1016/j.jacc.2013.09.068

[8] Iskandrian AE, Bateman TM, Belardinelli L, Blackburn B, Cerqueira MD, Hendel RC, et al. Adenosine versus regadenoson comparative evaluation in myocardial perfusion imaging: results of the ADVANCE phase 3 multicenter international trial. J Nucl Cardiol. 2007;14(5):645–58. 17826318 10.1016/j.nuclcard.2007.06.114

[9] Mahmarian JJ, Cerqueira MD, Iskandrian AE, Bateman TM, Thomas GS, Hendel RC, et al. Regadenoson induces comparable left ventricular perfusion defects as adenosine: a quantitative analysis from the ADVANCE MPI 2 trial. JACC Cardiovasc Imaging. 2009;2(8):959–68. 19679284 10.1016/j.jcmg.2009.04.011

[10] Navare SM, Mather JF, Shaw LJ, Fowler MS, Heller GV. Comparison of risk stratification with pharmacologic and exercise stress myocardial perfusion imaging: a meta-analysis. J Nucl Cardiol. 2004;11(5):551–61. 15472640 10.1016/j.nuclcard.2004.06.128

[11] Brinkert M, Reyes E, Walker S, Latus K, Maenhout A, Mizumoto R, et al. Regadenoson in Europe: first-year experience of regadenoson stress combined with submaximal exercise in patients undergoing myocardial perfusion scintigraphy. Eur J Nucl Med Mol Imaging. 2014;41(3):511–21. 24265072 10.1007/s00259-013-2619-0 PMC3913852

[12] Thomas GS, Prill NV, Majmundar H, Fabrizi RR, Thomas JJ, Hayashida C, et al. Treadmill exercise during adenosine infusion is safe, results in fewer adverse reactions, and improves myocardial perfusion image quality. J Nucl Cardiol. 2000;7(5):439–46. 11083192 10.1067/mnc.2000.108030

[13] Cerqueira MD, Nguyen P, Staehr P, Underwood SR, Iskandrian AE. Effects of age, gender, obesity, and diabetes on the efficacy and safety of the selective A2A agonist regadenoson versus adenosine in myocardial perfusion imaging integrated ADVANCE-MPI trial results. JACC Cardiovasc Imaging. 2008;1(3):307–16. 19356442 10.1016/j.jcmg.2008.02.003

[14] Leaker BR, O’Connor B, Hansel TT, Barnes PJ, Meng L, Mathur VS, et al. Safety of regadenoson, an adenosine A2A receptor agonist for myocardial perfusion imaging, in mild asthma and moderate asthma patients: a randomized, double-blind, placebo-controlled trial. J Nucl Cardiol. 2008;15(3):329–36. 18513639 10.1016/j.nuclcard.2008.02.009

[15] Thomas GS, Tammelin BR, Schiffman GL, Marquez R, Rice DL, Milikien D, et al. Safety of regadenoson, a selective adenosine A2A agonist, in patients with chronic obstructive pulmonary disease: a randomized, double-blind, placebo-controlled trial (RegCOPD trial). J Nucl Cardiol. 2008;15(3):319–28. 18513638 10.1016/j.nuclcard.2008.02.013

[16] Agarwal V, DePuey EG. Regadenoson and seizures: a real clinical concern. J Nucl Cardiol. 2014;21(5):869–70. 25150095 10.1007/s12350-014-9970-9

[17] Fukuda M, Suzuki Y, Hino H, Morimoto T, Ishii E. Activation of central adenosine A(2A) receptors lowers the seizure threshold of hyperthermia-induced seizure in childhood rats. Seizure. 2011;20(2):156–9. 21144776 10.1016/j.seizure.2010.11.012

[18] Page 2nd RL, Spurck P, Bainbridge JL, Michalek J, Quaife RA. Seizures associated with regadenoson: a case series. J Nucl Cardiol. 2012;19(2):389–91. 22002651 10.1007/s12350-011-9461-1

[19] Hage FG. Regadenoson for myocardial perfusion imaging: is it safe? J Nucl Cardiol. 2014;21(5):871–6. 24939324 10.1007/s12350-014-9922-4

[20] Pharmacovigilance Risk Assessment Committee (PRAC), Minutes of the meeting on 5–8 May 2014. http://www.ema.europa.eu/docs/en_GB/document_library/Minutes/2014/06/WC500169468.pdf. 2014. Accessed 7 July 2015.

[21] ICRP. Human alimentary tract model for radiological protection. ICRP Publication 100. A report of The International Commission on Radiological Protection. Ann ICRP. 2006;36(1-2):25–327. **iii**. 17188183 10.1016/j.icrp.2006.03.004

[22] ICRP. The 2007 Recommendations of the International Commission on radiological protection. ICRP publication 103. Ann ICRP. 2007;37(2-4):1–332. 10.1016/j.icrp.2007.10.003 18082557

[23] ICRP. Adult reference computational phantoms. ICRP Publication 110. Ann ICRP. 2009;39(2). 10.1016/j.icrp.2009.09.001 19897132

[24] Andersson M, Johansson L, Minarik D, Leide-Svegborn S, Mattsson S. Effective dose to adult patients from 338 radiopharmaceuticals estimated using ICRP biokinetic data, ICRP/ICRU computational reference phantoms and ICRP 2007 tissue weighting factors. EJNMMI Phys. 2014;1(1):9. 26501451 10.1186/2197-7364-1-9 PMC4545621

[25] Duvall WL, Croft LB, Ginsberg ES, Einstein AJ, Guma KA, George T, et al. Reduced isotope dose and imaging time with a high-efficiency CZT SPECT camera. J Nucl Cardiol. 2011;18(5):847–57. 21528422 10.1007/s12350-011-9379-7

[26] Herzog BA, Buechel RR, Katz R, Brueckner M, Husmann L, Burger IA, et al. Nuclear myocardial perfusion imaging with a cadmium-zinc-telluride detector technique: optimized protocol for scan time reduction. J Nucl Med. 2010;51(1):46–51. 20008999 10.2967/jnumed.109.065532

[27] Nkoulou R, Pazhenkottil AP, Kuest SM, Ghadri JR, Wolfrum M, Husmann L, et al. Semiconductor detectors allow low-dose-low-dose 1-day SPECT myocardial perfusion imaging. J Nucl Med. 2011;52(8):1204–9. 21810589 10.2967/jnumed.110.085415

[28] Oddstig J, Hedeer F, Jogi J, Carlsson M, Hindorf C, Engblom H. Reduced administered activity, reduced acquisition time, and preserved image quality for the new CZT camera. J Nucl Cardiol. 2013;20(1):38–44. 23143809 10.1007/s12350-012-9634-6

[29] Sharir T, Ben-Haim S, Merzon K, Prochorov V, Dickman D, Ben-Haim S, et al. High-speed myocardial perfusion imaging initial clinical comparison with conventional dual detector anger camera imaging. JACC Cardiovasc Imaging. 2008;1(2):156–63. 19356422 10.1016/j.jcmg.2007.12.004

[30] Lassmann M. The new EANM paediatric dosage card. Eur J Nucl Med Mol Imaging. 2008;35(9):1748. 18719912 10.1007/s00259-007-0572-5

[31] Buechel RR, Husmann L, Herzog BA, Pazhenkottil AP, Nkoulou R, Ghadri JR, et al. Low-dose computed tomography coronary angiography with prospective electrocardiogram triggering: feasibility in a large population. J Am Coll Cardiol. 2011;57(3):332–6. 21232672 10.1016/j.jacc.2010.08.634

[32] Maruyama T, Takada M, Hasuike T, Yoshikawa A, Namimatsu E, Yoshizumi T. Radiation dose reduction and coronary assessability of prospective electrocardiogram-gated computed tomography coronary angiography: comparison with retrospective electrocardiogram-gated helical scan. J Am Coll Cardiol. 2008;52(18):1450–5. 19017511 10.1016/j.jacc.2008.07.048

[33] Achenbach S, Marwan M, Ropers D, Schepis T, Pflederer T, Anders K, et al. Coronary computed tomography angiography with a consistent dose below 1 mSv using prospectively electrocardiogram-triggered high-pitch spiral acquisition. Eur Heart J. 2010;31(3):340–6. 19897497 10.1093/eurheartj/ehp470

[34] Fuchs TA, Stehli J, Bull S, Dougoud S, Clerc OF, Herzog BA, et al. Coronary computed tomography angiography with model-based iterative reconstruction using a radiation exposure similar to chest X-ray examination. Eur Heart J. 2014;35(17):1131–6. 24553723 10.1093/eurheartj/ehu053 PMC4006092

[35] DePuey EG. Advances in SPECT camera software and hardware: currently available and new on the horizon. J Nucl Cardiol. 2012;19(3):551–81. **quiz 85**. 22456968 10.1007/s12350-012-9544-7

[36] Garcia EV, Faber TL, Esteves FP. Cardiac dedicated ultrafast SPECT cameras: new designs and clinical implications. J Nucl Med. 2011;52(2):210–7. 21233190 10.2967/jnumed.110.081323

[37] Hutton BF. Developments in cardiac-specific SPECT imaging. Q J Nucl Med Mol Imaging. 2012;56(3):221–9. 22695334

[38] Einstein AJ, Blankstein R, Andrews H, Fish M, Padgett R, Hayes SW, et al. Comparison of image quality, myocardial perfusion, and left ventricular function between standard imaging and single-injection ultra-low-dose imaging using a high-efficiency SPECT camera: the MILLISIEVERT study. J Nucl Med. 2014;55(9):1430–7. 24982439 10.2967/jnumed.114.138222 PMC4486330

[39] Ben-Haim S, Almukhailed O, Neill J, Slomka P, Allie R, Shiti D, et al. Clinical value of supine and upright myocardial perfusion imaging in obese patients using the D-SPECT camera. J Nucl Cardiol. 2014;21(3):8. 10.1007/s12350-014-9853-0 24477404

[40] Chawla D, Rahaby M, Amin AP, Vashistha R, Alyousef T, Martinez HX, et al. Soft tissue attenuation patterns in stress myocardial perfusion SPECT images: a comparison between supine and upright acquisition systems. J Nucl Cardiol. 2011;18(2):281–90. 21234826 10.1007/s12350-010-9336-x

[41] Abbara S, Arbab-Zadeh A, Callister TQ, Desai MY, Mamuya W, Thomson L, et al. SCCT guidelines for performance of coronary computed tomographic angiography: a report of the Society of Cardiovascular Computed Tomography Guidelines Committee. J Cardiovasc Comput Tomogr. 2009;3(3):190–204. 19409872 10.1016/j.jcct.2009.03.004

[42] Delbeke D, Coleman RE, Guiberteau MJ, Brown ML, Royal HD, Siegel BA, et al. Procedure guideline for SPECT/CT imaging 1.0. J Nucl Med. 2006;47(7):1227–34. 16818960

[43] Utsunomiya D, Nakaura T, Honda T, Shiraishi S, Tomiguchi S, Kawanaka K, et al. Object-specific attenuation correction at SPECT/CT in thorax: optimization of respiratory protocol for image registration. Radiology. 2005;237(2):662–9. 16170014 10.1148/radiol.2372041387

[44] Dilsizian V, Bacharach SL, Beanlands RS, Bergmann SR, Delbeke D, Grople RJ, et al. PET myocardial perfusion and metabolism clinical imaging. http://www.asnc.org/imageuploads/ImagingGuidelinesPET. 2008. Accessed 7 July 2015.

[45] Lehner S, Sussebach C, Todica A, Uebleis C, Brunner S, Bartenstein P, et al. Influence of SPECT attenuation correction on the quantification of hibernating myocardium as derived from combined myocardial perfusion SPECT and (1)(8)F-FDG PET. J Nucl Cardiol. 2014;21(3):578–87. 24633501 10.1007/s12350-014-9882-8

[46] Einstein AJ, Johnson LL, Bokhari S, Son J, Thompson RC, Bateman TM, et al. Agreement of visual estimation of coronary artery calcium from low-dose CT attenuation correction scans in hybrid PET/CT and SPECT/CT with standard Agatston score. J Am Coll Cardiol. 2010;56(23):1914–21. 21109114 10.1016/j.jacc.2010.05.057 PMC3040452

[47] Burkhard N, Herzog BA, Husmann L, Pazhenkottil AP, Burger IA, Buechel RR, et al. Coronary calcium score scans for attenuation correction of quantitative PET/CT 13N-ammonia myocardial perfusion imaging. Eur J Nucl Med Mol Imaging. 2010;37(3):517–21. 19774376 10.1007/s00259-009-1271-1

[48] Schepis T, Gaemperli O, Koepfli P, Ruegg C, Burger C, Leschka S, et al. Use of coronary calcium score scans from stand-alone multislice computed tomography for attenuation correction of myocardial perfusion SPECT. Eur J Nucl Med Mol Imaging. 2007;34(1):11–9. 16896667 10.1007/s00259-006-0173-8

[49] Miller JM, Rochitte CE, Dewey M, Arbab-Zadeh A, Niinuma H, Gottlieb I, et al. Diagnostic performance of coronary angiography by 64-row CT. N Engl J Med. 2008;359(22):2324–36. 19038879 10.1056/NEJMoa0806576

[50] Vavere AL, Arbab-Zadeh A, Rochitte CE, Dewey M, Niinuma H, Gottlieb I, et al. Coronary artery stenoses: accuracy of 64-detector row CT angiography in segments with mild, moderate, or severe calcification—a subanalysis of the CORE-64 trial. Radiology. 2011;261(1):100–8. 21828192 10.1148/radiol.11110537 PMC3176425

[51] ACR–NASCI–SPR Practice guideline for the performance and interpretation of cardiac computed tomography. http://www.acr.org/~/media/f4720a18f03b4a26a0c9c3cc18637d87.pdf. 2011. Accessed 7 July 2015.

[52] Lembcke A, Schwenke C, Hein PA, Knobloch G, Durmus T, Hamm B, et al. High-pitch dual-source CT coronary angiography with low volumes of contrast medium. Eur Radiol. 2014;24(1):120–7. 23949727 10.1007/s00330-013-2988-6

[53] Hoffmann U, Ferencik M, Cury RC, Pena AJ. Coronary CT angiography. J Nucl Med. 2006;47(5):797–806. 16644750

[54] Schroeder S, Achenbach S, Bengel F, Burgstahler C, Cademartiri F, de Feyter P, et al. Cardiac computed tomography: indications, applications, limitations, and training requirements: report of a Writing Group deployed by the Working Group Nuclear Cardiology and Cardiac CT of the European Society of Cardiology and the European Council of Nuclear Cardiology. Eur Heart J. 2008;29(4):531–56. 18084017 10.1093/eurheartj/ehm544

[55] ACR Committee on Drugs and Contrast Media. ACR Manual on Contrast Media, Version 9, 2013. http://www.acr.org/quality-safety/resources/~/media/37D84428BF1D4E1B9A3A2918DA9E27A3.pdf/. 2013. Accessed 7 July 2015.

[56] Boellaard R, Delgado-Bolton R, Oyen WJ, Giammarile F, Tatsch K, Eschner W, et al. FDG PET/CT: EANM procedure guidelines for tumour imaging: version 2.0. Eur J Nucl Med Mol Imaging. 2015;42(2):328–54. 25452219 10.1007/s00259-014-2961-x PMC4315529

[57] Schopp JG, Iyer RS, Wang CL, Petscavage JM, Paladin AM, Bush WH, et al. Allergic reactions to iodinated contrast media: premedication considerations for patients at risk. Emerg Radiol. 2013;20(4):299–306. 23430296 10.1007/s10140-012-1081-9

[58] Thomsen HS. Contrast media safety-an update. Eur J Radiol. 2011;80(1):77–82. 21856102 10.1016/j.ejrad.2010.12.104

[59] Cochran ST, Bomyea K, Sayre JW. Trends in adverse events after IV administration of contrast media. AJR Am J Roentgenol. 2001;176(6):1385–8. 11373197 10.2214/ajr.176.6.1761385

[60] Mortele KJ, Oliva MR, Ondategui S, Ros PR, Silverman SG. Universal use of nonionic iodinated contrast medium for CT: evaluation of safety in a large urban teaching hospital. AJR Am J Roentgenol. 2005;184(1):31–4. 15615946 10.2214/ajr.184.1.01840031

[61] Wang CL, Cohan RH, Ellis JH, Caoili EM, Wang G, Francis IR. Frequency, outcome, and appropriateness of treatment of nonionic iodinated contrast media reactions. AJR Am J Roentgenol. 2008;191(2):409–15. 18647910 10.2214/AJR.07.3421

[62] Katayama H, Yamaguchi K, Kozuka T, Takashima T, Seez P, Matsuura K. Adverse reactions to ionic and nonionic contrast media. A report from the Japanese Committee on the Safety of Contrast Media. Radiology. 1990;175(3):621–8. 2343107 10.1148/radiology.175.3.2343107

[63] Kaufmann PA. Cardiac hybrid imaging: state-of-the-art. Ann Nucl Med. 2009;23(4):325–31. 19360454 10.1007/s12149-009-0245-5

[64] Raff GL, Abidov A, Achenbach S, Berman DS, Boxt LM, Budoff MJ, et al. SCCT guidelines for the interpretation and reporting of coronary computed tomographic angiography. J Cardiovasc Comput Tomogr. 2009;3(2):122–36. 19272853 10.1016/j.jcct.2009.01.001

[65] Cerqueira MD, Weissman NJ, Dilsizian V, Jacobs AK, Kaul S, Laskey WK, et al. Standardized myocardial segmentation and nomenclature for tomographic imaging of the heart. A statement for healthcare professionals from the Cardiac Imaging Committee of the Council on Clinical Cardiology of the American Heart Association. Circulation. 2002;105(4):539–42. 11815441 10.1161/hc0402.102975

[66] Javadi MS, Lautamaki R, Merrill J, Voicu C, Epley W, McBride G, et al. Definition of vascular territories on myocardial perfusion images by integration with true coronary anatomy: a hybrid PET/CT analysis. J Nucl Med. 2010;51(2):198–203. 20080895 10.2967/jnumed.109.067488

[67] Gaemperli O, Bengel FM, Kaufmann PA. Cardiac hybrid imaging. Eur Heart J. 2011;32(17):2100–8. 21406437 10.1093/eurheartj/ehr057

[68] Gaemperli O, Schepis T, Valenta I, Husmann L, Scheffel H, Duerst V, et al. Cardiac image fusion from stand-alone SPECT and CT: clinical experience. J Nucl Med. 2007;48(5):696–703. 17475956 10.2967/jnumed.106.037606

[69] Santana CA, Garcia EV, Faber TL, Sirineni GK, Esteves FP, Sanyal R, et al. Diagnostic performance of fusion of myocardial perfusion imaging (MPI) and computed tomography coronary angiography. J Nucl Cardiol. 2009;16(2):201–11. 19156478 10.1007/s12350-008-9019-z PMC3086676

[70] Slomka PJ, Baum RP. Multimodality image registration with software: state-of-the-art. Eur J Nucl Med Mol Imaging. 2009;36 Suppl 1:S44–55. 19104803 10.1007/s00259-008-0941-8

[71] Slomka PJ. Software approach to merging molecular with anatomic information. J Nucl Med. 2004;45 Suppl 1:36s–45. 14736834

[72] Budoff MJ, Dowe D, Jollis JG, Gitter M, Sutherland J, Halamert E, et al. Diagnostic performance of 64-multidetector row coronary computed tomographic angiography for evaluation of coronary artery stenosis in individuals without known coronary artery disease: results from the prospective multicenter ACCURACY (Assessment by Coronary Computed Tomographic Angiography of Individuals Undergoing Invasive Coronary Angiography) trial. J Am Coll Cardiol. 2008;52(21):1724–32. 19007693 10.1016/j.jacc.2008.07.031

[73] Meijboom WB, Meijs MF, Schuijf JD, Cramer MJ, Mollet NR, van Mieghem CA, et al. Diagnostic accuracy of 64-slice computed tomography coronary angiography: a prospective, multicenter, multivendor study. J Am Coll Cardiol. 2008;52(25):2135–44. 19095130 10.1016/j.jacc.2008.08.058

[74] Husmann L, Tatsugami F, Aepli U, Herzog BA, Valenta I, Veit-Haibach P, et al. Prevalence of noncardiac findings on low dose 64-slice computed tomography used for attenuation correction in myocardial perfusion imaging with SPECT. Int J Cardiovasc Imaging. 2009;25(8):859–65. 19662511 10.1007/s10554-009-9490-x

[75] Rispler S, Keidar Z, Ghersin E, Roguin A, Soil A, Dragu R, et al. Integrated single-photon emission computed tomography and computed tomography coronary angiography for the assessment of hemodynamically significant coronary artery lesions. J Am Coll Cardiol. 2007;49(10):1059–67. 17349885 10.1016/j.jacc.2006.10.069

[76] Sato A, Nozato T, Hikita H, Miyazaki S, Takahashi Y, Kuwahara T, et al. Incremental value of combining 64-slice computed tomography angiography with stress nuclear myocardial perfusion imaging to improve noninvasive detection of coronary artery disease. J Nucl Cardiol. 2010;17(1):19–26. 19777317 10.1007/s12350-009-9150-5

[77] Pazhenkottil AP, Nkoulou RN, Ghadri JR, Herzog BA, Buechel RR, Kuest SM, et al. Prognostic value of cardiac hybrid imaging integrating single-photon emission computed tomography with coronary computed tomography angiography. Eur Heart J. 2011;32(12):1465–71. 21320906 10.1093/eurheartj/ehr047

[78] van Werkhoven JM, Schuijf JD, Gaemperli O, Jukema JW, Boersma E, Wijns W, et al. Prognostic value of multislice computed tomography and gated single-photon emission computed tomography in patients with suspected coronary artery disease. J Am Coll Cardiol. 2009;53(7):623–32. 19215839 10.1016/j.jacc.2008.10.043

[79] Pazhenkottil AP, Nkoulou RN, Ghadri JR, Herzog BA, Kuest SM, Husmann L, et al. Impact of cardiac hybrid single-photon emission computed tomography/computed tomography imaging on choice of treatment strategy in coronary artery disease. Eur Heart J. 2011;32(22):2824–9. 21804107 10.1093/eurheartj/ehr232 PMC3214723

[80] Fiechter M, Ghadri JR, Wolfrum M, Kuest SM, Pazhenkottil AP, Nkoulou RN, et al. Downstream resource utilization following hybrid cardiac imaging with an integrated cadmium-zinc-telluride/64-slice CT device. Eur J Nucl Med Mol Imaging. 2012;39(3):430–6. 22143224 10.1007/s00259-011-1999-2

